# Predictive role of neuron-specific enolase and S100-β in early neurological deterioration and unfavorable prognosis in patients with ischemic stroke

**DOI:** 10.1515/med-2024-1043

**Published:** 2024-12-11

**Authors:** Ruishu Jiang, Youlian Lai

**Affiliations:** Department of Neurology, The Second Hospital of Longyan, No.8, Shuangyang West Road, Beicheng, Xinluo District, Longyan, Fujian, 364000, China; Department of Neurology, The Second Hospital of Longyan, Longyan, Fujian, 364000, China

**Keywords:** neuron-specific enolase, S100-β, acute ischemic stroke, early neurological deterioration, prognosis

## Abstract

**Background:**

We aimed to assess whether neuron-specific enolase (NSE) and S100-β levels are associated with early neurological deterioration (END) in patients with acute ischemic stroke (AIS).

**Methods:**

We conducted a prospective study between March 2022 and October 2023 in 286 patients with AIS. Serum NSE and S100-β levels on admission and at 24 and 48 h after stroke onset were measured using electrochemiluminescence immunoassays. Outcomes included END events within 48 h of admission and unfavorable neurological outcomes at 3 months.

**Results:**

Patients with END had higher serum NSE and S100-β levels. Patients with poor prognosis had higher serum NSE and S100-β levels. Serum NSE (on admission) was an independent biomarker for END in AIS patients and for unfavorable recovery at 3 months. In addition, serum S100-β was an independent biomarker of unfavorable recovery after 3 months in patients with AIS.

**Conclusion:**

Serum NSE on admission and S100-β at 48 h of stroke onset may serve as biomarkers of short-term clinical outcome in patients with AIS. Elevated serum NSE and S100-β levels may be useful tools to predict prognosis in patients with AIS.

## 1 Introduction

Stroke is the second leading cause of adult disability and death worldwide [1]. Strokes are generally divided into two types: ischemic and hemorrhagic strokes, with ischemic strokes accounting for 85% of all strokes [2]. As it is strongly associated with disability and cognitive dysfunction [3], it has a significant impact on public health. Despite progress in disease prevention and acute management in China, changes in demographics are expected to increase the burden of stroke [4,5,6]. Some patients experience early neurological deterioration (END) in the acute phase, with a prevalence of up to one-third [7,8]. Neurological deterioration that occurs early or subacutely may interfere with functional recovery in several studies [9,10,11]. Therefore, rapid prediction of clinical outcomes in acute ischemic stroke (AIS) is essential to improve the prognosis of patients.

Currently, serum biochemical markers are becoming a hot topic in stroke research, since they may become a simple and rapid way to assess irreversible injuries at the bedside. Neuron-specific enolase (NSE) is a neuronal glycolytic enzyme that is abundant in gray matter neurons of the brain and is involved in axonal transport [12]. In recent years, it has been reported about NSE as a marker of AIS [13]. In addition, in the cerebrospinal fluid of the brain, NSE can be used as a biomarker for the detection of neonatal ischemic–hypoxic encephalopathy [14]. The mechanism of its release is mainly due to hypoxia and ischemia of brain tissue, neurons are damaged, necrotic, and disintegrated, resulting in damage to the blood–brain barrier, and subsequently, NSE in disintegrated neurons diffuses into the cerebrospinal fluid and is released into the blood [15]. As brain damage aggravates, the number of dead and disintegrated neurons increases, and the damage to the blood–brain barrier is more severe, which leads to an increase in the level of NSE in the blood. It can be seen that NSE reflects the severity of brain injury and changes in the condition through its unique release mechanism and concentration changes in AIS, and this elevated level of NSE is closely related to the severity of brain injury and can be used as an auxiliary marker for the diagnosis of ischemic-related diseases. However, the level of serum NSE detection is also affected by many factors such as detection system, genotype, geographic location, and lifestyle.

S100-β is a small-molecule calcium-binding protein that does not cross the blood–brain barrier into the bloodstream under normal conditions and leaks out of the cerebrospinal fluid and into the peripheral bloodstream when the blood–brain barrier is compromised [16]. S100-β is secreted extracellularly by astrocytes and maintained at low concentrations, thereby promoting synaptic growth, neuronal survival, and to some extent neuroprotection [17]. In contrast, under the stimulation of pathological factors, astrocytes produce and release large amounts of S100-β, which has neurotoxic effects [18]. Some vascular-related diseases, such as atherosclerosis [19], hypercholesterolemia [20], and acute coronary syndromes [21], are associated with S100-β. In addition, S100-β has been reported to be used as a biomarker for the diagnosis of cerebral ischemia and ischemic stroke [22,23]. High concentrations of S100-β can contribute to the process of brain injury by exacerbating brain edema and promoting inflammation through neurotoxic mechanisms. For example, in an *in vitro* study, astrocytes release large amounts of S100β protein, and the protein mediates the production of reactive oxygen species (ROS) through RAGE-dependent mediation, which exerts toxic effects on neurons in a manner that activates the ROS/RAS/ERK1/2 pathway [24]. Elevated S100-β has been reported to be associated with neurological deficits [25].

To the best of our knowledge, so far, the role of circulating NSE and S100-β in IS has been addressed more as markers for adjunctive diagnosis, and the prognosis of circulating NSE and S100-β levels in such patients has not been reported. Therefore, the aim of this study was to investigate the role of serum NSE and S100-β in clinical outcomes, including END and neurological recovery at 3 months in AIS patients.

## 2 Materials and methods

### 2.1 Study population

Between March 2022 and October 2023, patients who met the clinical diagnosis of AIS were prospectively recruited. They were confirmed by imaging results and hospitalized within 12–14 h of symptom onset. Exclusion criteria were as follows: (1) age <18 years; (2) discharge or transfer within 3 days of admission; (3) previous severe pulmonary disease, renal failure, hepatic failure, previous traumatic brain injury, or concomitant malignant neoplastic disease; (4) evidence of a second stroke episode, non-ischemic stroke, or infectious brain disease during the study period; and (5) loss to follow-up. All subjects signed an informed consent. The study was approved by The Second Hospital of Longyan ethics committee (No. 202103FJ-30).

### 2.2 Baseline data and assessment of clinical outcomes

Detailed demographic and clinical data were collected by trained neurological clinicians after admission. These data included age, gender, body mass index (BMI), smoking, cardiovascular risk factors, baseline stroke severity, stroke subtype, and blood pressure level. All patients had an imaging diagnosis of ischemic stroke on admission by CT or MRI. Baseline stroke severity was assessed by the National Institutes of Health Stroke Scale (NIHSS) [26]. Stroke subtypes were etiologically classified using the ORG 10172 Trial of Acute Stroke Treatment (TOAST) [27]. In addition, patients who received intravenous thrombolysis or bridging therapy were included in the study.

Clinical outcomes included END occurrence and neurological recovery outcomes 3 months after stroke onset. Baseline neurological deficits were assessed on a daily basis for 48 h (1–3 times per day) using the NIHSS. END was defined as an increase of ≥4 points in NIHSS score (non-hemorrhagic results) within 48 h of admission [28]. Follow-up was performed over 3 months using the modified Rankin rating scale (mRS). Functional outcomes were categorized as favorable (mRS 0–2) and unfavorable (mRS 3–6) [29].

### 2.3 Blood sampling

The samples were collected in sterile vacuum collection tubes without anticoagulant. Samples were left at room temperature for 2 h or overnight at 4°C and centrifuged at 2,000× *g* for 15 min. The supernatant was collected and stored at −80°C.

### 2.4 Laboratory test

An automated biochemical analyzer Beckman Coulter AU5800 (Beckman Coulter, Brea, CA, USA) was applied to measure fasting blood glucose (FBG), homocysteine, serum total cholesterol, serum triglycerides, high-density lipoprotein-C, low-density lipoprotein-C, D-dimer (D-D), and high-sensitivity C-protein response (hs-CRP).

NSE activity and S100-β were determined by electrochemiluminescence immunoassay on a Roche Modular E 170 analyzer (Roche, Mannheim, Germany). The lower limit of NSE was 0.05 μg/L, and the upper limit was 370 μg/L. The intra-assay and inter-assay variations were less than 5.0% in both the 0.90 and 95.1 μg/L ranges. The lower limit for S100-β was 0.005 μg/L and the upper limit was 39 μg/L. The intra-assay variation and inter-assay variation for the ranges of 0.26 and 2.25 μg/L were less than 3.0%.

Normal values of NSE and S100-β were assessed by internal laboratory normal values established by a control group of 32 healthy volunteers.

### 2.5 Data analysis

Categorical variables were expressed as frequencies (%), and chi-square or Fisher’s exact test was used for comparisons between groups. For numerical variables, Shapiro–Wilk analysis was used to test the normality of the data. Variables that conformed to a normal distribution were expressed as mean ± standard deviation, and differences between groups were compared using an independent Student’s *t*-test. Skewed distribution variables were expressed as medians (25th–75th percentiles [interquartile range {IQR}]) and compared using Mann–Whitney *U* test between groups or Kruskal–Wallis test among three groups. *P*-values were then corrected by the Bonferroni method. Spearman bivariate correlation analysis was used while *P* values were adjusted by multiple testing through FDR and Benjamini Hochberg correction. Logistic regression was used to screen for the occurrence of END and unfavorable prognosis. The predictive validity of biomarkers for the occurrence of END and unfavorable prognosis in patients with AIS was assessed using receiver operating characteristic curves (ROC) and the area under the ROC curve (AUC). Data analysis was performed with SPSS software 22.0 and plotting was performed with GraphPad Prism 9.5.0. *P* values below 0.05 (2-tailed) were considered statistically significant.


**Informed consent:** Written informed consent was provided by all patients prior to the study start.
**Ethical approval:** The present study was approved by the Ethics Committee of The Second Hospital of Longyan (No. 202103FJ-30). All procedures were performed in accordance with the ethical standards of the Institutional Review Board and The Declaration of Helsinki and its later amendments or comparable ethical standards.

## 3 Results

### 3.1 General clinical characteristics and biochemical parameters of the patient

**Figure 1:**
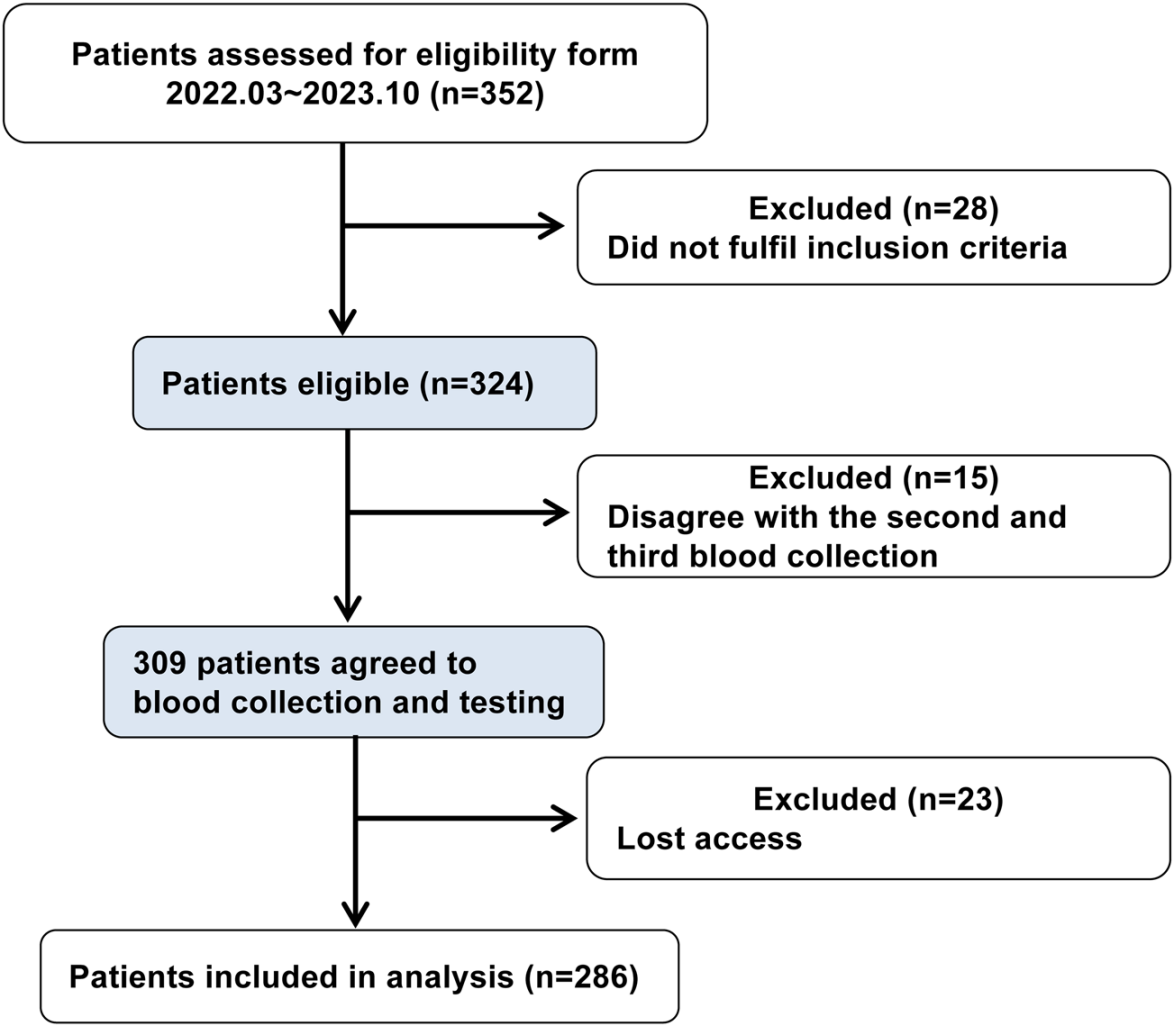
Clinical process.

Between March 2022 and October 2023, 352 patients were enrolled in the study. Twenty-three patients were lost to follow-up, resulting in the inclusion of 286 individuals in the study. A total of 10 (3.5%) patients died within 3 months of the stroke event. Figure 1 provides a flow chart of the study population.

Baseline characteristics and biochemical indices of patients are given in Table 1. The mean age of patients with AIS was 62.3 ± 11.7 years and 62.3% (180/286) were male. END events occurred in 18.3% of cases (53/286). Patients were divided into two subgroups (with END and without END) based on the NIHSS score at 48 h of admission. The median baseline NIHSS score for patients with END was 8 IQR (4–10). Of the stroke-related vascular risk diseases, diabetes accounted for nearly half the proportion of patients with END, that is, 43.4% (23/53). FBG was higher in patients with END (*P* = 0.015). In addition, no differences in other baseline information were observed between patients with and without END (*P* > 0.005).

**Table 1:** General clinical characteristics and biochemical parameters of the patients

Characteristics	With END (***n*** = 53)	Without END (*n* = 233)	P value	Unfavorable (*n* = 137)	Favorable (*n* = 149)	P value
Age, years	64 [53–72]	60 [50–72]	0.254	63 [56–73]	60 [53–71]	0.025
Gender, *n* (%)			0.143			0.077
Male	38 (71.7)	142 (60.94)		79 (57.66)	101 (67.79)	
Female	15 (28.3)	91 (39.06)		58 (42.34)	48 (32.21)	
BMI (kg/m^2^)	24.3 ± 3.2	23.6 ± 4.5	0.127	23.8 ± 3.6	24.3 ± 3.8	0.562
Tobacco use, *n* (%)	10 (18.87)	53	0.539	25 (18.25)	38	0.139
Disease factors, *n* (%)						
Hypertension	13 (24.53)	40 (17.17)	0.095	33 (24.09)	20 (13.42)	0.02
Diabetes	23 (43.4)	68 (29.18)	0.045	56 (40.88)	35 (23.49)	0.002
Dyslipemia	9 (16.98)	44 (18.88)	0.204	28 (20.44)	25 (16.78)	0.426
ACS	5 (9.43)	25 (10.73)	0.781	15 (10.95)	13 (8.72)	0.527
TOAST classification			0.792			0.088
Cardio-embolism	16 (30.19)	58 (24.89)		42 (30.66)	32 (21.48)	
Atherosclerosis	8 (33.96)	75 (32.19)		40 (29.20)	53 (35.57)	
Small vessel occlusion	4 (7.55)	20 (8.58)		15 (10.95)	9 (6.04)	
Undetermined etiology	15 (28.30)	80 (34.33)		40 (29.20)	55 (36.91)	
END	—	—	—	38 (27.74)	17 (11.41)	<0.001
SBP (mmHg)	159 ± 16	148 ± 29	0.069	163 ± 17	152 ± 21	0.035
DBP (mmHg)	88.5 ± 7.6	86.6 ± 9.5	0.492	88.7 ± 8.2	87.4 ± 8.6	0.752
NIHSS (on admission)	8 [4–10]	4 [3.0–7]	<0.001	8 [4–10]	4 [3.0–8]	<0.001
Δ NIHSS	4 [4–5]	2 [1–3]	<0.001	3 [2–4]	2 [1–3]	0.001
mRS	5 [3–5]	2 [2–4]	0.01	5 [3–5]	1 [0–2]	<0.001
IV rt-PA, *n* (%)	15 (28.30)	53 (22.75)	0.391	22 (16.06)	41 (27.52)	0.02
Bridging therapy, *n* (%)	2 (3.77)	12 (5.15)	0.675	9 (6.57)	5 (3.36)	0.208
FBG (mmol/L)	7.7 [6.5–9.3]	6.7 [5.2–8.1]	0.015	7.0 [5.9–8.6]	6.3 [5.3.–8.0]	0.017
D-D (ng/mL)	219 [190.6–235.3]	195.1 [176.3–213.3]	0.035	210.3 [203.3–221.3]	203.4 [195.3–214.3]	0.021
hs-CRP (mg/L)	5.36 [2.35–8.35]	3.24 [0.78–6.05]	<0.001	4.18 [1.58–7.38]	3.42 [1.06–6.84]	0.015

BMI, body mass index; ACS, acute coronary syndrome; END, early neurological deterioration; SBP, systolic blood pressure; DBP, diastolic blood pressure; NHISS, The National Institutes of Health Stroke Scale; Δ NIHSS = NIHSS score (at 48 h)－NIHSS on admission; IV rt-PA, intravenous recombinant tissue plasminogen activator; FBG, fasting blood glucose; D-D, D-dimer; hs CRP, high-sensitive C-reactive protein. Continuous data are shown as median (interquartile range, IQR) or mean ± SD, and categorical data are expressed as *N* (%). Categorical values were compared using the chi-square test. Student’s *t*-test or Mann–Whitney test was used to assess the differences between the two groups. *P* < 0.05 was statistically significant.

Patients with AIS were categorized into two subgroups, unfavorable prognosis, and favorable prognosis, based on their neurological recovery 3 months after stroke onset. In terms of clinical characteristics, patients with unfavorable prognoses had older age (*P* = 0.025) and higher levels of SBP (*P* = 0.035) and FBG (*P* = 0.017). Moreover, patients with unfavorable prognosis were more likely to have hypertension (*P* = 0.020) and diabetes (*P* = 0.002). Of the 53 patients who had an END, 38 had an adverse prognostic event. In addition, a higher proportion of patients with a favorable prognosis, 27.52% (41/149) received intravenous thrombolysis compared with the unfavorable prognosis group. In terms of biochemical indices, we observed high levels of serum D-D and hs-CRP in patients with END and in those with unfavorable prognosis (*P* < 0.005).

### 3.2 Serum NSE and S100-β levels in patients

**Figure 2:**
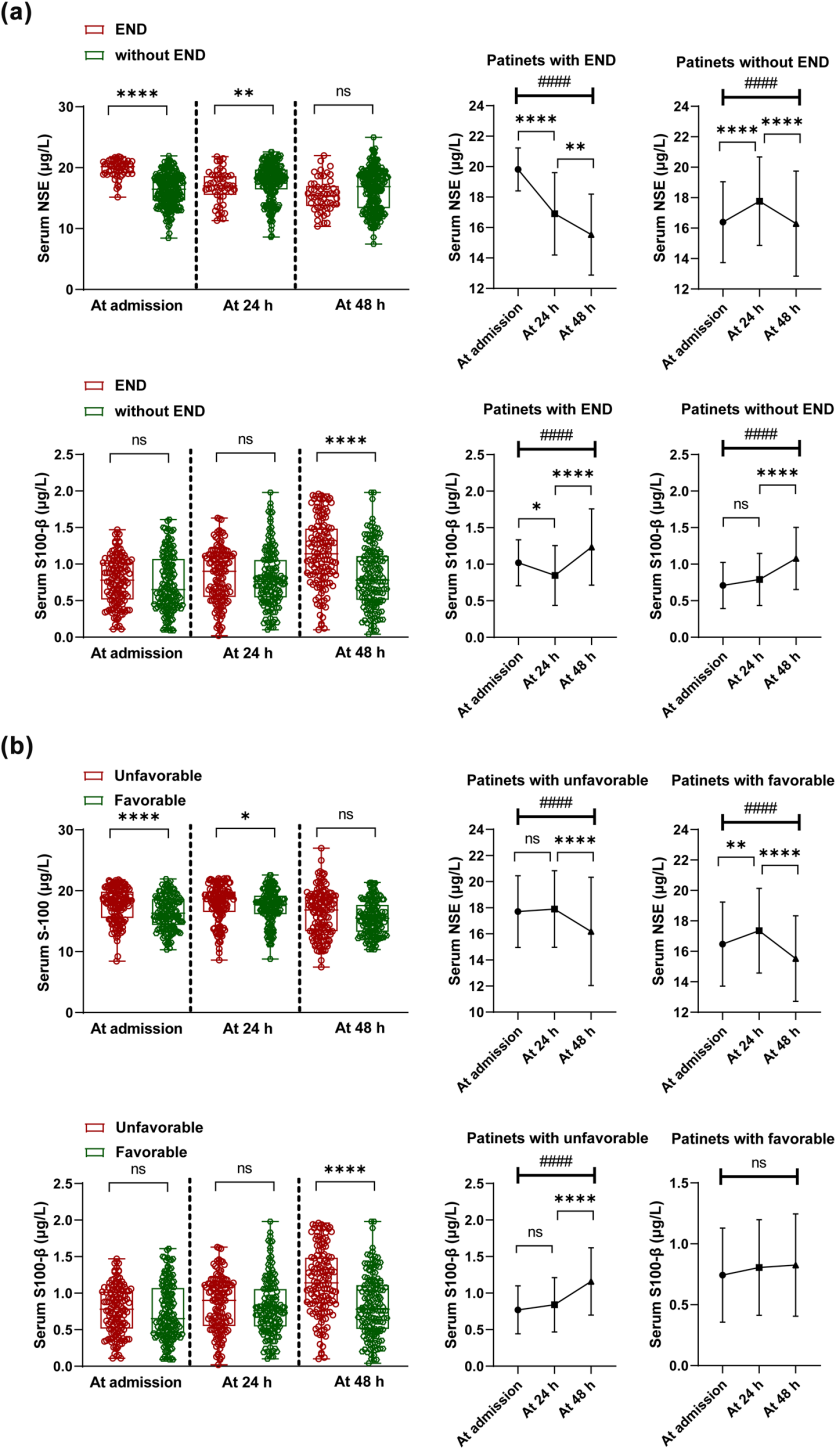
Serum NSE and S100-β levels. (a) Serum NSE and S100-β levels in patients with END and without END on admission, 24 h and 48 h after stroke onset. (b) Serum NSE and S100-β levels on admission, 24 h and 48 h after stroke in patients with unfavorable and favorable prognosis. ****P < 0.0001, **P = 0.01, *P < 0.05, ns, *P* > 0.05. *P* < 0.05 is statistically significant.

The serum NSE and S100-β levels in the control group were 13.35 [11.14–15.3] μg/L and 0.36 [0.21–0.66] μg/L, respectively, which were significantly higher than those in AIS patients with serum NSE levels of 17.50 [15.02–19.21] and S100-β levels of 0.72 [0.46–1.03] (*P* < 0.0001). Serum NSE (on admission), NSE (at 24 h), and S100-β (at 48 h) levels were higher in patients with END than those without END (*P* < 0.05). In addition, patients with END had the highest serum NSE and S100-β levels on admission and at 48 h of stroke onset, respectively (Figure 2a). Serum NSE (on admission), NSE (at 24 h), and S100-β (at 48 h) levels were higher in patients with unfavorable prognosis than in patients with favorable prognosis (*P* < 0.05). Serum NSE and S100-β levels were highest in patients with an unfavorable prognosis on admission and at 48 h of stroke onset, respectively (Figure 2b).

Next, we performed correlation analyses using serum levels at both time points for variables associated with END or adverse outcomes. Table 2 shows that serum NSE (on admission) had a moderate strong positive correlation with NIHSS, mRS, and hs-CRP (0.5 > rs > 0.30, *P* < 0.05) and a weak positive correlation with NIHSS (on admission) (rs = 0.293, *P* < 0.05). S100-β (at 48 h) was found to have a moderately strong correlation with mRS (rs = 0.402, *P* < 0.05) and a weak correlation with NIHSS (on admission), NIHSS, and D-D (rs < 0.30, *P* < 0.05).

**Table 2:** Correlation analysis of serum NSE and S100-B levels and identification of variables associated with the occurrence of END or adverse outcomes

	NIHSS (on admission)	D NIHSS	mRS	D-D	hs CRP
NSE (on admission)	0.293*	0.342*	0.366*	0.154	0.385*
S100-β (at 48 h)	0.275*	0.135*	0.402*	0.242*	0.185

Spearman’s correlation analysis was used to determine the relationship between variables and biomarker levels associated with adverse outcomes. *P < 0.05.

### 3.3 Independent correlates of clinical outcomes

**Figure 3:**
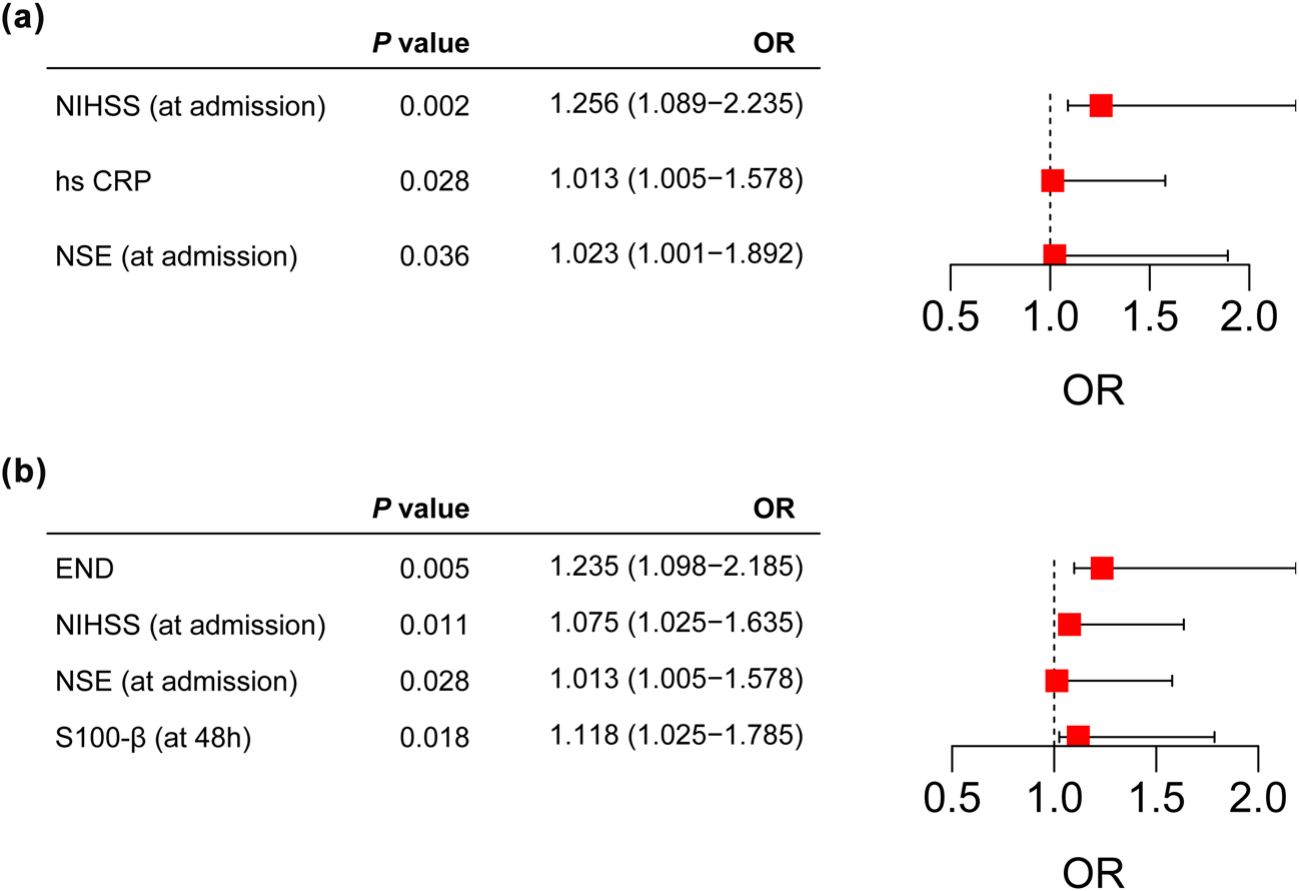
Logistic analysis between serum NSE on admission and serum S100-β 48 h after stroke onset and END or adverse outcomes. (a) Independent correlates of END in patients. (b) Independent correlates of unfavorable prognosis. *P* < 0.05 is statistically significant.

Due to the restricted number of patients in the END group, some categorical variables such as hypertension and diabetes were closely clinically correlated with continuous numerical variables (e.g., blood pressure values, and blood glucose values). Therefore, we used only categorical variables (hypertension and diabetes) as screening variables when it comes to these variables. Binary logistic regression incorporated variables with *P* values less than 0.05 in the univariate analyses as candidate variables, and age was corrected as a confounder. As shown in Figure 3a, NIHSS score (on admission), hs-CRP, and serum NSE (on admission) were independent correlates of END in patients with AIS. As shown in Figure 3b, END, NIHSS score (on admission), serum NSE (on admission), and S100-β (at 48 h) were independent correlates of unfavorable neurological recovery at 3 months in AIS patients.

### 3.4 Predictive performance of serum NSE and S100-β

Table 3 shows the accuracy of clinical factors and markers in predicting END and 3-month prognosis in patients with AIS. Based on the ROC curve, serum NSE (on admission) level of 18.83 μg/L showed the best sensitivity (88.36%) and specificity (42.35%) for predicting END in patients, with an AUC of 0.713 (95% CI, 0.669–0.772; *P* = 0.003). Based on the AUC, the predictive value of NSE (on admission) was lower than that of NHISS score (on admission) (*P* = 0.0101) and similar to that of hs-CRP. We configured a combined logistic regression model and found that the AUC of NSE combined with NIHSS on admission (model 1) was not significantly different from the AUC of NIHSS score (*P* = 0.109), but NSE combined with hs-CRP (model 2) statistically significantly increased the AUC of hs-CRP to 0.725 (*P* = 0.002).

**Table 3:** Accuracy of markers in predicting the occurrence of END events and 3-month prognosis in patients with AIS

Prediction	Sensitivity (%)	Specificity (%)	AUC	95% CI	*P* value
**Predicting the occurrence of END events**					
NIHSS	68.38	89.3	0.806	0.732–0.865	<0.001
hs CRP	52.36	65.42	0.605	0.515–0.664	0.018
NSE (on admission)	88.36	42.35	0.713	0.664–0.772	0.003
Model 1	66.32	92.35	0.835	0.765–0.892	<0.001
Model 2	72.35	70.36	0.725	0.685–0.798	0.001
**Predicting unfavorable events 3 months after stroke onset**					
NIHSS	48.69	60.36	0.605	0.569–0.675	0.01
END	90.36	75.36	0.793	0.758–0.864	<0.001
NSE (on admission)	65.38	40.35	0.585	0.529–0.662	0.012
S100-β (at 48 h)	73.69	55.35	0.689	0.599–0.742	0.003
Model A	66.69	60.36	0.823	0.765–0.858	<0.001
Model B	74.36	65.36	0.735	0.685–0.816	<0.001

AUC, area under the curve. 95% CI, confidence interval. Model 1, NSE combined with NIHSS on admission; model 2, NSE combined with hs-CRP on admission. Model A, S100-β combined with END at 48 h of stroke onset. Model B, S100-β combined with NHISS at 48 h of stroke onset. *P*-value less than 0.05 was considered statistically significant.

For the 3-month prognosis of AIS patients, serum NSE (on admission) level of 17.05 μg/L provided the best sensitivity (65.38%) and specificity (40.35%) according to the ROC curve, with an AUC of 0.585 (95% CI, 0.529–0.662; *P* = 0.003). Serum S100-β (at 48 h) of 0.815 μg/L provided the best sensitivity (63.69%) and specificity (45.35%) for predicting prognosis, with an AUC of 0.659 (95% CI, 0.579–0.742; *P* = 0.003). Based on AUC, NSE (on admission) (*P* = 0.005) and S100-β (at 48 h) (*P* = 0.012) were predicted to be lower than END and did not differ from the NHISS score (both *P* > 0.05). In addition, S100-β at 48 h of stroke onset was predicted to be higher than serum NSE on admission (*P* = 0.025). We found that the AUC of S100-β combined with END at 48 h of stroke onset (model A) was not significantly different from the AUC of END (*P* = 0.379), but that S100-β combined with NHISS score (model B) statistically significantly increased the AUC of NHISS score to 0.735 (*P* = 0.016).

## 4 Discussion

This prospective observational study suggests that serum NSE on admission and S100-β at 48 h of stroke onset serve as independent prognostic biomarkers. Elevated serum NSE and S100-β levels may be useful tools to predict prognosis in patients with AIS.

Few attempts have been made to investigate the clinical significance of serum NSE and S100-β levels over time in patients with AIS. A previous study has focused on the relationship between NSE and S100-β levels and short-term (7–10 days) functional outcomes after AIS [13]. In addition, serum NSE and S100-β levels increase with increasing NIHSS scores for 3 consecutive days after admission in patients with AIS [13]. Similar to the above results, we found a positive correlation between serum NSE and S100-β levels and NIHSS score. In contrast, the above study demonstrated that serum NSE and S100-β were consistently elevated in patients with good or poor prognosis, peaked in the first 3 days, and began to decline on day 4. Our study showed that in the prognostic subgroup, serum NSE reached its highest concentration at 24 h of stroke onset and decreased thereafter. Differences in sampling time (24 h after onset versus on admission) and NIHSS scores in the study population may be partly responsible for this. The mean NIHSS score on admission reached close to 10 in the previous study population, whereas the mean NIHSS score in our study was 7. The presence of patient heterogeneity requires further and more detailed information for comparison. In addition, several studies reported that D-D and hs-CRP were significantly associated with adverse stroke events, such as stroke recurrence, END events, and post-stroke death [30,31]. We observed, as expected, that D-D and hs-CRP levels were significantly increased in patients who developed END and unfavorable functional prognoses.

In recent years, there has been a growing body of research on brain-derived biomarkers, based on the concept that all biomarkers from neuronal tissue can be used to measure the severity of brain injury. Continuous measurements are feasible, and the possibility of bedside detection methods offers a promising prospect. NSE is a cytoplasmic protein involved in axonal transport and is found mainly in neurons and neuroendocrine cells [12]. When axonal damage occurs, NSE is upregulated to maintain homeostasis *in vivo* [32]. Therefore, NSE is considered to be a sensitive marker that responds to the degree of neuronal damage. When brain white matter injury occurs, astrocytes release S100-β, which eventually enters the cerebrospinal fluid and bloodstream [33]. It has been demonstrated that NSE and S100-β are highly sensitive quantitative indicators that increase neuronal injuries such as craniocerebral injury, stroke, cerebral hemorrhage, and cardiac arrest [34,35,36]. We believe that ischemia in the brain leads to dysfunction of glial cells. NSE and S100-β enter the bloodstream through the blood–brain barrier. In the current study, it is reasonable to expect elevated serum NSE and S100-β levels in stroke patients compared with controls.

In previous studies, most of them reported the correlation between NSE and S100-β levels at a single time point and patients’ clinical characteristics, such as infarct size [37] and degree of neurological deficit [38]. However, changes in NSE and S100-β levels at multiple time points after stroke onset were not observed. In our study, we first determined that there were significant differences in serum NSE and S100-β between patients with and without END and between patients with favorable and unfavorable prognoses at different time intervals. Elevated levels of S100-β on admission and NSE 48 h after stroke onset were found to be associated with END events and poor prognosis. Several studies have shown that elevated D-D and hs-CRP are predictors of poor prognosis in ischemic stroke [39,40]. Using bivariate correlation analysis, we confirmed that NSE on admission and S100-β level at 48 h of stroke onset was significantly associated with hs-CRP and D-D, respectively. However, only hs-CRP was observed to be an independent correlate in predicting the risk of END events in patients with AIS. This suggests a potential mechanistic correlation between NSE and hs-CRP on admission. In our study, serum NSE on admission was an independent risk factor for END events and unfavorable prognosis at 3 months in AIS patients. Most studies have shown that END is an independent correlate of short-term poor recovery in patients with IS [8,41]. In addition, serum NSE combined with hs-CRP improved the predictive efficacy of hs-CRP for END events in AIS patients. Finally, serum NSE on admission and S100-β level at 48 h of stroke onset were independent predictors of functional recovery at 3 months in patients with AIS. Compared with serum NSE, S100-β at 48 h of stroke onset had a higher predictive value for AIS prognosis.

Currently, there are no studies reporting the classical mechanisms of NSE transport at the cell surface. However, NSE increases after spinal cord injury and aggravates ischemic neuropathic injury by activating fibrinogen activity and matrix metalloproteinases, thereby promoting and maintaining a pro-inflammatory microenvironment [40]. High S100-β concentration may interfere with the normal homeostasis of intracellular calcium ions, leading to pathological phenomena such as calcium overload and increasing the likelihood of neuronal injury. Therefore, we believe that the elevated levels of NES and S100-β are mainly due to the release of neuronal damage into the bloodstream, while high concentrations of NES and S100-β are synergistically involved in promoting the release of inflammatory factors and mediating neurotoxicity, inducing infiltration of inflammatory cells and apoptosis and necrosis of neuronal cells, which aggravate the progression of brain injury and ultimately lead to END events. To the best of our knowledge, serum S100-β is associated with cerebral white matter lesions, and in clinical practice, patients with ischemic stroke develop cerebral white matter microstructural damage [42]. Cerebral white matter, especially subcortical white matter, has been reported to be associated with long-term neurological function after brain injury [43]. Thus, in patients with AIS, S100-β may not only be associated with brain damage leading to the activation and release of astrocytes into the bloodstream but may also be involved in alterations of cerebral white matter microstructure, which may further affect neurological function. Therefore, the prognostic value of S100-β in IS patients is higher than that of NSE. However, more relevant studies are needed to confirm this.

The results of this study should be interpreted with caution. First, we are assuming that both serum NSE and S100-β originate from the damaged brain, and it is uncertain whether their circulating levels are the same as the changes in the central nervous system. In addition, it is not known whether there are significant fluctuations in NSE and S100-β that impair neurological function and thus affect the prognosis of the patient after AIS. In the correlation risk assessment, for confounding factors, we mainly chose age and gender to be included in the analysis. Although these final results did not show that these factors were associated with patient prognosis, it is necessary to perform other statistical methods and techniques to construct more accurate predictive models. Despite the fact that we included intravenous thrombolytic therapy in the multifactorial logistic analysis for correction, NSE and S100-β remained independent factors influencing prognosis. However, the effect of intravenous thrombolytic therapy on changes in NSE and S100-β levels cannot be excluded. The results of this study apply only to the cohort studied, and generalization of the findings to other populations (e.g., patients with depression, Alzheimer’s disease, or roles in other brain disorders) should be done with caution. The present study only explored the correlation between changes in serum NSE and S100-β levels and the occurrence of END and poor prognosis in patients with IS from admission to 48 h later and did not delve deeper into the pathophysiologically relevant mechanisms involved. In an *in vitro* study, acute stress affects the disease process by activating sensitive brain regions [44]. Therefore, it is necessary to explore the pathophysiologically relevant mechanisms of NSE and S100-β in different sensitive brain regions in IS patients in future studies.

## 5 Conclusion

Our study found elevated serum NSE and S100-β levels to be a useful tool for predicting END events and unfavorable prognosis in patients with AIS. The current findings suggest that serum NSE and S100-β have clinical significance and represent potential new therapeutic targets for AIS.
